# Fast and Robust Diffusion Posterior Sampling for MR Image Reconstruction Using the Preconditioned Unadjusted Langevin Algorithm

**DOI:** 10.1002/mrm.70416

**Published:** 2026-05-10

**Authors:** Moritz Blumenthal, Tina Holliber, Jonathan I. Tamir, Martin Uecker

**Affiliations:** ^1^ Institute of Biomedical Imaging Graz University of Technology Graz Austria; ^2^ Department of Radiology Boston Children's Hospital, Harvard Medical School Boston USA; ^3^ Chandra Family Department of Electrical Engineering University of Texas at Austin Austin USA; ^4^ Department of Diagnostic Medicine, Dell Medical School University of Texas at Austin Austin USA; ^5^ BioTechMed‐Graz Graz Austria

**Keywords:** Bayesian reconstruction, diffusion posterior sampling, image reconstruction, MRI, parallel imaging

## Abstract

**Purpose:**

The Unadjusted Langevin Algorithm (ULA) in combination with diffusion models can generate high quality MRI reconstructions with uncertainty estimation from highly undersampled k‐space data. However, sampling methods such as diffusion posterior sampling (DPS) or likelihood annealing suffer from long reconstruction times and the need for parameter tuning. The purpose of this work is to develop a robust sampling algorithm with fast convergence.

**Theory and Methods:**

In the reverse diffusion process used for sampling the posterior, the exact likelihood is multiplied with the diffused prior at all noise scales. To overcome the issue of slow convergence, preconditioning is used. The method is trained on fastMRI data and tested on retrospectively undersampled brain data of a healthy volunteer.

**Results:**

For posterior sampling in Cartesian and non‐Cartesian accelerated MRI the new approach outperforms annealed sampling and DPS in terms of reconstruction speed and sample quality.

**Conclusion:**

The proposed exact likelihood with preconditioning enables rapid and reliable posterior sampling across various MRI reconstruction tasks without the need for parameter tuning.

## 1 Introduction

In recent years, diffusion models have shown impressive results in image generation by providing an approach for sampling from a high‐dimensional probability distribution. In a Bayesian setting, they can be used as a learned prior to perform MRI reconstruction even from highly undersampled k‐space data by sampling the posterior probability distribution [1, 2, 3, 4]. This Bayesian approach to image reconstruction has several key advantages over other deep learning methods: (i) by decoupling the measurement model from the prior, no retraining is necessary when the measurement operator changes (e.g., due to different sampling trajectories, coil arrays, motion, field inhomogeneities); (ii) the measurement noise level uniquely determines the relative weighting of prior and likelihood, that is no tuning of a regularization parameter is required; and (iii) uncertainty maps computed based on the full posterior can show when the reconstruction becomes unreliable due to an insufficient amount of data [3].

While sampling the unconditional prior can be done efficiently using a reverse diffusion process based on a learned series of smoothed prior distributions [5, 6, 7], efficient sampling of the posterior distribution based on a realistic acquisition model such as SENSE [8] is still a challenging problem. Directly learning the posterior [9], or various modifications of the likelihood term were proposed [10, 11] to improve the sampling of the posterior distribution in a reverse diffusion process, including annealed [1] or noisy [12, 13] likelihoods. These methods are relatively slow and require a careful choice of step size and reverse‐diffusion noise schedule to achieve good results. Crucially, these parameters must be tuned for different measurement models, thus losing some of the purported flexibility offered by the Bayesian approach.

For example, Chung et al. showed that using the correct measurement noise level for their DPS [12] method yields poor results. To achieve good reconstruction results, they introduced a heuristic for reweighting the likelihood and prior, which requires tuning of an additional hyperparameter for different measurement models or noise levels and ultimately samples from a distribution that does not correspond to the true posterior.

In this work, we observe that the practical difficulties encountered when sampling the true posterior distribution are caused by the ill‐conditioning of the problem. For convergence, small step sizes and many noise scales must be used which then prevents effective sampling. We tackle this problem by preconditioning: Degrees of freedom that correspond to large singular values of the SENSE model, i.e., which are strongly restricted by the measurements, are refined with small updates, while degrees of freedom corresponding to small singular values are refined with large updates allowing them to traverse the probability mass given by the learned prior distribution more freely. Our approach can then be used with a fixed pre‐chosen step size and thus eliminate the need to tune it for different acquisitions. The weighting of the likelihood and prior is completely determined by the noise level of the measurements, which is known from an adjustment scan or can be estimated from the image background.

In addition, we show that our method yields faster convergence compared to the annealed likelihood [1] and DPS [12] approach. In particular, this affects non‐Cartesian sampling, where the highly non‐uniform sampling of k‐space leads to a high condition number of the SENSE model and, hence, to slow convergence. This is issue is partially addressed by the Decomposed Diffusion Sampler (DDS) [14], which performs sampling efficiently in Krylow subspaces, but introduces a new hyperparameter for the relative weighting of the likelihood and prior.

We tested the proposed method on both Cartesian and non‐Cartesian radial brain data, showing consistently better reconstruction quality and lower computation time compared to annealing and DPS without any need for parameter tuning.

## 2 Theory

### 2.1 Bayesian Reconstruction

MRI reconstruction can be formulated as the inverse problem

(1) y=Ax+n,n∼ℂN(0,I)

with k‐space data y, the SENSE model A, image x and white complex Gaussian noise n of unit variance (after prewhitening, normalization, and adapting the coil sensitivities accordingly). From the Bayesian perspective, the posterior probability density p(x|y) is given by

(2) $$p\left(x|y\right)=\frac{p\left(y|x\right)p\left(x\right)}{p\left(y\right)}\propto \exp\left(-∥y-Ax{∥}_{2}^{2}+\log p\left(x\right)\right),$$

with the prior p(x) and the likelihood p(y|x). From the posterior distribution, point estimators such as the maximum a posteriori (MAP) estimate, which corresponds to conventional image reconstruction with regularization, or the minimum mean square error (MMSE) estimate can be computed. To estimate the MMSE, multiple samples can be drawn from the posterior using the unadjusted Langevin algorithm (ULA) and averaged. For complex valued random vectors, the ULA update reads

(3) $$x^{k+1}=x^{k}+\gamma \;\nabla_{\bar{x}}\log p\left(x^{k}|y\right)+\sqrt{2\gamma }z^{k}\;z^{k}∼\mathbb{C}N\left(0,I\right),$$

where $\nabla_{\bar{x}}\log p\left(x|y\right)$ is the score function of the posterior distribution and $\nabla_{\bar{x}}$ is the complex conjugate gradient operator of Wirtinger calculus [15]. The corresponding complex likelihood score is given by

(4) $$\nabla_{\bar{x}}\log p\left(y|x\right)=A^{H}\left(y-Ax\right).$$

For convergence and to reduce the discretization bias, the step size γ must be sufficiently small compared to the inverse Lipschitz $L^{-1}$ constant of the posterior score [16, 17]. The score $\nabla_{\bar{x}}\log p\left(x_{0}\right)$ of a smoothed version of the prior can be obtained from a training data set of images using denoising score matching (DSM) [5].

### 2.2 Posterior Sampling

The goal of posterior sampling is to draw samples from the posterior distribution in Equation 2, where the prior distribution $p\left(x_{0}\right)$ is learned. Because direct sampling of a high‐dimensional multi‐modal distribution is not practically possible, a reverse diffusion process is used. For sampling of the prior [5, 6], this can be done by successive sampling of a series of priors $p\left(x_{t}\right)$ smoothed by convolutions with complex Gaussians of variance $\sigma_{t}^{2}$, starting from t=1 with a simple complex Gaussian distribution of variance $\sigma_{1}^{2}=\sigma_{\max}^{2}$ that can be sampled directly until the lowest noise scale $\sigma_{0}=\sigma_{\min}$ is reached, where the learned score is assumed to approximate the true prior score well.

A straightforward idea is to formulate a diffusion process for the posterior in the same way as for the prior by adding Gaussian noise [18]. To be able to use the learned prior, at each time t the diffused posterior $p\left(x_{t}|y\right)$ should factorize into the diffused prior $p\left(x_{t}\right)$ and the conditional probability for measuring y when observing a perturbed sample $x_{t}$. By marginalization over the unknown noiseless images x one arrives at

(5) $$p^{\text{diffused}}\left(y|x_{t}\right)=\int p\left(y|x\right)p\left(x|x_{t}\right)dx.$$

We call this approach *Diffused Posterior*. However, this likelihood term needs to be approximated in practice, as it makes use of the posterior for the denoising problem $p\left(x|x_{t}\right)$, which is simpler than the full posterior but still intractable. This problem has led to various approximations for the term $p^{\text{diffused}}\left(y|x_{t}\right)$. Jalal et al. [1] introduced a weighting term $\kappa_{t}$ added to the data noise during the diffusion process:

(6) $$p^{\text{annealed}}\left(y|x_{t}\right)\propto \exp\left(-\frac{1}{1+\kappa_{t}}∥y-Ax_{t}{∥}_{2}^{2}\right),$$

where $\kappa_{t}\to 0$ for decreasing noise scale $\sigma_{t}$ which we call *Annealed Likelihood*. Chung et al. [12] used the score output by the network to calculate the expectation $E\left[x_{0}|x_{t}\right]$ in order to approximate $p\left(x|x_{t}\right)\approx \delta \left(x-E\left[x_{0}|x_{t}\right]\right)$ at every noise scale, i.e.,

(7) $$p^{\mathrm{DPS}}\left(y|x_{t}\right)\propto \exp\left(-∥y-AE\left[x_{0}|x_{t}\right]{∥}_{2}^{2}\right).$$

While diffusing the posterior distribution is conceptually straightforward but practically challenging, an alternative approach is to use a different reverse process so long as the correct posterior distribution is obtained at t=0. This insight was first described in Sohl‐Dickstein et al. [19], in which the diffusion process was modified by multiplication with a function (e.g., the likelihood term). Hence, the learned prior distribution $p\left(x_{t}\right)$ can be multiplied by a modified likelihood term $p\left(y|x_{t}\right)$, which smoothly varies with t and is equal to the true likelihood p(y|x) at t=0. Here, we investigate the use of the original likelihood term in Equation 2, i.e.,

(8) $$p^{\text{exact}}\left(y|x_{t}\right)\propto \exp\left(-∥y-Ax_{t}{∥}_{2}^{2}\right),$$

for the whole diffusion process, at every noise scale. Previously, this has been seen as a inacurrate approximation of the diffused likelihood in Equation 5. However, here we use the original likelihood term from Equation 2 to construct a diffusion process not based on Equation 5 that, when understood as a different process, does not require any approximation. Hence, we call the likelihood *Exact Likelihood*.

**FIGURE 1:**
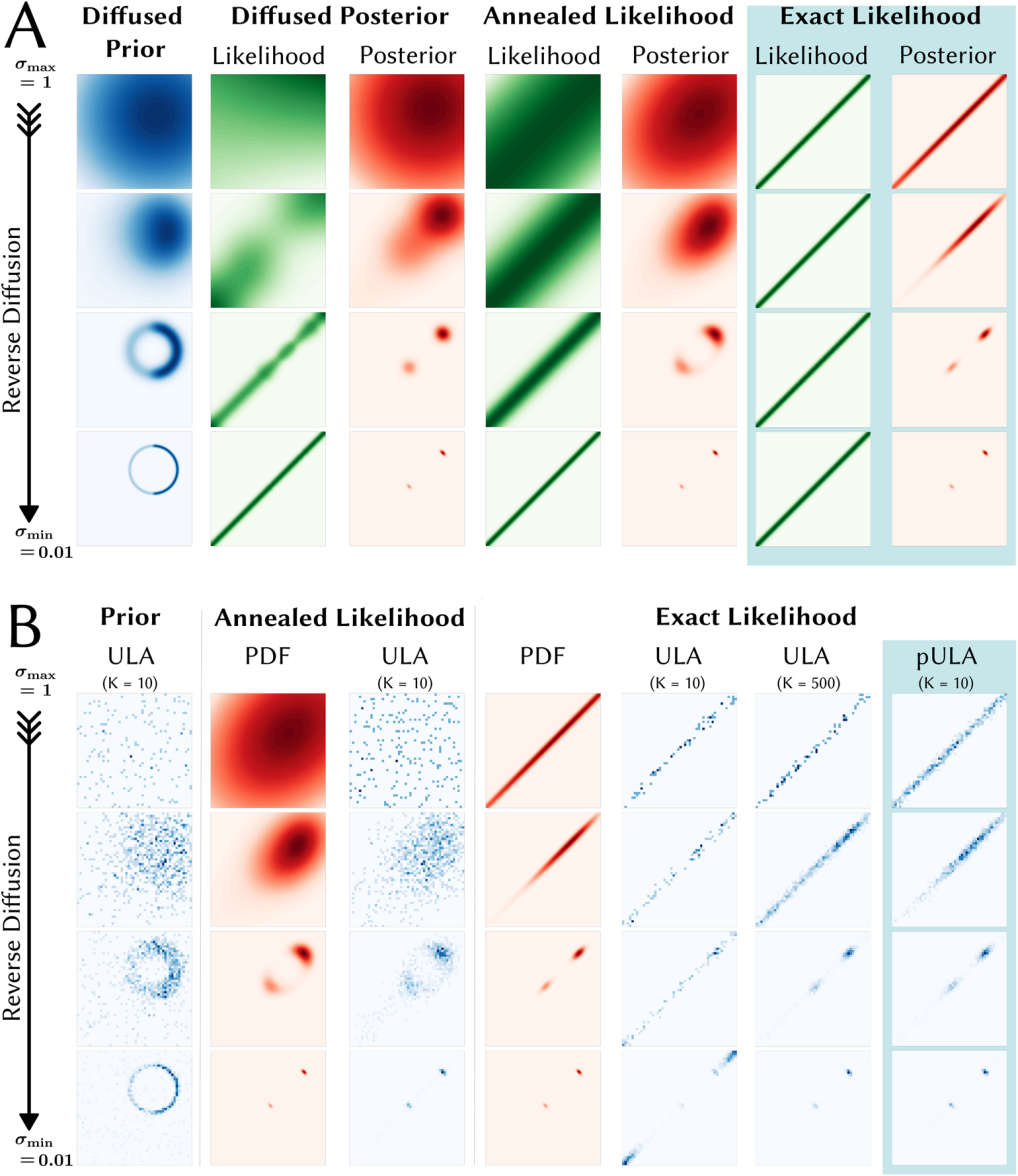
A: Analytical diffusion process for a 2D toy model with a 1D linear measurement $A={\left(1-1\right)}^{T}$. The prior distribution is a mixture of 2D Gaussians forming a circle, which models the data manifold. Likelihood modifications corresponding to the diffused posterior and annealing are compared to the exact likelihood. All methods have the same posterior distribution at the minimum noise scale. B: Samples of the same 2D toy model with annealed (left) and exact (right) likelihood sampled with ULA for 10 and 500 iterations and pULA for 10 iterations. Samples are shown for noise levels σ=[1,0.2,0.05,0.01].

A brief discussion how this approach can be translated to the variance preserving formulation of diffusion models is provided in Supporting Information. To motivate this choice, we show the effect of the choice of the likelihood for a 2D toy model in Figure 1A that can be analytically computed similar to [9].

In contrast to the *Annealed Likelihood* or *Diffused Likelihood*, the *Exact Likelihood* approach smoothes the posterior distribution only in directions that are not or weakly constrained by the measurement. Importantly, all three likelihoods lead to the same, correct posterior distribution at the lowest noise level.

### 2.3 Preconditioning of ULA

A naive use of the *Exact Likelihood* method is impractical because some directions are then constrained by the data already at high noise scales, implying a large Lipschitz constant corresponding to the maximum eigenvalue of $A^{H}A$ in the posterior score, exacerbated in non‐Cartesian sampling. This then prevents the use of larger step sizes, leading to slow convergence. To solve this issue, we propose a preconditioned ULA (pULA) with the update rule [20, 21, 22, 23]:

(9) $$x_{t}^{k+1}=x_{t}^{k}+\gamma M_{t}\left[A^{H}\left(y-Ax_{t}^{k}\right)+\nabla_{\bar{x}}\log p_{t}\left(x_{t}^{k}\right)\right]+\sqrt{2\gamma }z^{k}\;z^{k}∼\mathbb{C}N\left(0,M_{t}^{-1}\right),$$

with a preconditioning matrix $M_{t}$ specifically adapted to linear inverse problems, i.e., $M_{t}={\left(A^{H}A+\sigma_{t}^{-2}I\right)}^{-1}$. Here, the superscript denotes the k th Langevin update per diffusion time t. At t=1, the reverse‐diffusion process is started with a sample from the posterior distribution corresponding to a flat Gaussian prior with variance $\sigma_{1}^{2}=\sigma_{\max}^{2}$, i.e.,

(10) $$x_{t=1}^{k=0}∼\mathbb{C}N\left(M_{1}\;A^{H}y,M_{1}^{-1}\right).$$

At each noise level $\sigma_{t}$, we perform K Langevin steps of pULA before the noise level is reduced, and pULA is initialized with the last sample from the higher noise level.

Our choice of the precondition matrix $M_{t}$ is based on the following observation: The preconditioner should approximate the inverse Hessian of the negative log‐posterior density. The Hessian of the negative exact log‐likelihood is given by $A^{H}A$ and we approximated the Hessian of the learned negative log prior by $\sigma_{t}^{-2}I$, which corresponds to a Gaussian prior with variance $\sigma_{t}^{2}$. In combination with the likelihood, the preconditioner allows larger updates in directions of small singular values of A, that is, those which are only weakly determined by the measurements. In these directions, the preconditioner effectively yields a step size proportional to the variance of the diffusion noise $\sigma_{t}^{2}$ as empirically found to work well for prior sampling [5].

The initialization and update rule of pULA require drawing samples z from the distribution $\mathbb{C}N\left(0,M_{t}^{-1}\right)$. Those can be drawn efficiently using [24].

(11) $$z=M_{t}\left(A^{H}n_{1}+\sigma_{t}^{-1}n_{2}\right)\;\left(\begin{array}{c} n_{1}\\ n_{2} \end{array}\right)∼\mathbb{C}N\left(0,I\right),$$

similar to the pseudo replica method [25]. For the initialization, the mean can be added to the zero‐mean sample. Inserting Equation 11 into Equation 9, the update of pULA for the posterior score can be written as

(12) $$\begin{array}{ll} x_{t}^{k+1} & =x_{t}^{k}+\gamma M_{t}\left[A^{H}\left(y+\sqrt{\frac{2}{\gamma }}n_{1}^{k}-Ax_{t}^{k}\right)\right.\\ & \left.\;\;+\nabla_{\bar{x}}\log p\left(x_{t}^{k}\right)+\sqrt{\frac{2}{{\gamma \sigma }_{t}^{2}}}n_{2}^{k}\right],\;\;\left(\begin{array}{c} n_{1}^{k}\\ n_{2}^{k} \end{array}\right)∼\mathbb{C}N\left(0,I\right). \end{array}$$

In this form, it is apparent that the pULA update requires only one application of $M_{t}$, which can be performed using the conjugate gradient (CG) method without explicitly forming the preconditioning matrix.

## 3 Methods

### 3.1 Data Processing and Network Training

For training, we used images reconstructed from the fully sampled k‐space data of the multi‐coil train folder of the fastMRI brain dataset [26] that have a 320×320 matrix size. To be consistent with the inference pipeline, the k‐space data were pre‐whitened using a noise covariance matrix estimate from the background patches of the fully sampled coil images. The data were then compressed to 12 virtual coils, and coil sensitivities were estimated with a low‐resolution version of NLINV [27]. Using the corresponding reconstruction, a scaling for the k‐space was estimated such that the final reconstruction is normalized to have a 99 percentile pixel magnitude of one. A foreground mask for the images was estimated using ESPIRiT [28]. Data for which this mask extends to the image boundary in the phase encoding direction were assumed to be corrupted, for example by motion, and removed. After this step, the training dataset contains around 32 000 of the original 36 000 samples. To suppress background noise, the final images for training were then reconstructed using FISTA with a small $\ell_{1}$‐Wavelet regularization. Using these high quality images, we trained a conditional denoising U‐Net with conditional residual blocks [29] using Adam (learningrate=0.0001, batchsize=32 and epochs=100) implemented in BART [30]. The sigma schedule was an exponential decay from $\sigma_{\max}=100$ to $\sigma_{\min}=0.01$. From the denoising U‐Net, the score network is obtained using Tweedie's formula [25, 31].

### 3.2 MRI Data Acquisition

MRI data of a healthy volunteer was acquired on a 3 T scanner (Magnetom Vida, Siemens Healthineers, Erlangen, Germany) after obtaining written informed consent and with approval of the local ethics committee. We acquired fully sampled multi‐slice Cartesian T2‐weighted (TR=6000ms, TE=98ms, ΔTE=9.82ms, $\mathrm{FA}=150^{{}^{\circ}}$, ETL=16, $\mathrm{BW}=223\;\mathrm{Hz}\;{\mathrm{px}}^{-1}$) and T1‐weighted (TR=600ms, TE=6.70ms, ΔTE=6.69ms, $\mathrm{FA}=140^{{}^{\circ}}$, ETL=2, $\mathrm{BW}=391\;\mathrm{Hz}\;{\mathrm{px}}^{-1}$) brain data with a 20‐channel head coil using a Turbo Spin Echo sequence.

In addition, a radial T1‐weighted scan was acquired using a stack‐of‐stars FLASH sequence with a RAGA [32] sampling scheme (TR=8.0ms, TE=3.32ms, $\mathrm{FA}=10^{{}^{\circ}}$, voxel size: $0.8\times 0.8\times 3\;{\mathrm{mm}}^{3}$, $\mathrm{BW}=780\;\mathrm{Hz}\;{\mathrm{px}}^{-1}$). Two repetitions with 987 spokes each were acquired, i.e., two RAGA full frames. All datasets had a FoV of 250mm and matrix size of 320×320 pixels.

### 3.3 Numerical Experiments

We first tested the exact likelihood approach for a real‐valued 2D toy model with a linear measurement operator $A={\left(1-1\right)}^{T}$ and an analytical prior defined by a Gaussian mixture model. pULA with the exact likelihood was compared to ULA with exact and annealed likelihood, where in total N=101 noise scales were used with $\sigma_{\max}=1$ and $\sigma_{\min}=0.01$.

Then, the proposed exact likelihood method was compared to an $\ell_{1}$‐Wavelet regularized parallel imaging reconstruction, DPS and the annealed likelihood approach on a slice of the T2‐weighted dataset. For this, the fully sampled k‐space data were pre‐whitened based on noise extracted from a background patch of the fully sampled coil images. The data were then retrospectively undersampled with both equispaced and randomized undersampling masks (acceleration 4 and 12) with a 16‐line auto‐calibration region. Similar to the processing of the training data, the undersampled data were coil compressed to 12 virtual channels, coils were estimated with NLINV, a foreground mask was computed with ESPIRiT, and a normalization scale was computed from the low‐resolution NLINV reconstruction. Instead of scaling the k‐space data, we absorb this normalization constant in the coil sensitivities, such that the k‐space data stays white with unit variance noise and the scaling of the corresponding image remains consistent with normalization used for the training images.

The sampling techniques were implemented in BART. For the annealed likelihood, we choose the annealing parameter $\kappa_{t}$ such that

(13) $$\frac{1}{1+\kappa_{t}}={\left(\frac{\sigma_{\max}^{-2}}{\lambda_{\max}\left(A^{H}A\right)}\right)}^{t},$$

where $\lambda_{\max}\left(A^{H}A\right)$ is the maximum eigenvalue of $A^{H}A$, which we estimate using power iterations. At t=1, this choice balances the Lipschitz constants of the likelihood and the prior scores. For ULA of the annealed likelihood (aULA), we chose the step size $\gamma =\gamma_{\text{base}}{\left[{\left(1+\kappa_{t}\right)}^{-1}\lambda_{\max}\left(A^{H}A\right)+\sigma_{t}^{-2}\right]}^{-1}$ with a base scaling $\gamma_{\text{base}}=0.5$ in all experiments. For pULA, we use the same step size for all noise levels, namely, γ=0.5. The implementation of DPS is detailed in Supporting Information.

For the diffusion reconstruction of the T2‐weighted dataset, we exponentially reduced the diffusion noise level $\sigma_{t}$ from $\sigma_{\max}=10$ to $\sigma_{\min}=0.01$. Ten samples were drawn and averaged using aULA, DPS and pULA with exact likelihood. N=60 noise levels were used for aULA and pULA with K=8 (aULA) and K=4 (pULA) Langevin iterations per noise level. The preconditioner was applied with $N_{\mathrm{CG}}=10$ CG iterations using a warmstarting strategy detailed in Supporting Information. For DPS, we used the predictor sampling [29] with the same amount of network evaluations compared to the exact likelihood approach. The weighting of the DPS likelihood was set to $\zeta^{\prime }=0.2$, which yields best results for 12× acceleration. If not stated otherwise, the same parameters were used for all other experiments. In addition, we tested all methods starting at $\sigma_{\max}=1$ and $\sigma_{\max}=0.1$ and correspondingly reduced the number of noise levels to N=40 and N=20, respectively. The regularization parameter for the undersampled $\ell_{1}$‐Wavelet reconstruction was chosen by a grid search to obtain the best PSNR with respect to the fully sampled reconstruction. For all reconstructions, the time per sample was measured. All images were normalized by multiplication with the RSS of the coils to obtain an RSS scaling of the final reconstructions. Afterward, PSNR and SSIM values and error maps were computed with magnitude images relative to the fully sampled $\ell_{1}$‐Wavelet reconstruction after multiplying all images with the foreground mask.

To investigate the robustness of the method under substantially different experimental conditions, we performed three additional experiments. First, the number of virtual coils used for sampling/reconstruction of the T1‐weighted dataset was decreased to 4 or 1 after the pre‐processing described above, i.e., the full pre‐processing was performed using 12 virtual coils. Second, we performed sampling of the T2‐weighted dataset with three‐fold increased noise level. For DPS a grid search was conducted to determine the likelihood weighting $\zeta^{\prime }$ with optimal PSNR. Third, we performed reconstructions of the radial data, where we reduced the number of radial spokes from 987 spokes to 98, 49, and 24 spokes. To discard data during the transition towards the FLASH steady state, we used the data of the second frame only. The radial data was processed similar to the Cartesian data, but first an inverse FFT in partition direction was performed to allow for slice‐wise processing. The gradient delays were corrected with RING [33]. To compute the ESPIRiT foreground mask, k‐space data was gridded first, whereas coil sensitivities were directly estimated from the radial data with NLINV. In all cases, an $\ell_{1}$‐Wavelet reconstruction was also performed.

All numerical experiments have been performed on a system with an AMD EPYC 9334 CPU and an Nvidia H100 GPU (80 GB HBM3, SXM). We record and compare total runtime for all algorithms (c.f. Supporting Information).

## 4 Results

Figure 1B shows the 2D toy example of the sampling results for the reverse diffusion process comparing the annealed and the exact likelihood method for (p)ULA. In total 1000 samples were drawn. The annealed posterior adapts during the diffusion process, whereas the exact posterior does not change. For time zero, all methods converge to the correct posterior distribution. However, there are different convergence speeds. For the exact likelihood, ULA needs a small step size and therefore more iterations (K=500) to converge to the correct distribution. pULA with exact likelihood shows faster convergence, similar to the annealed approach (K=10).

**FIGURE 2:**
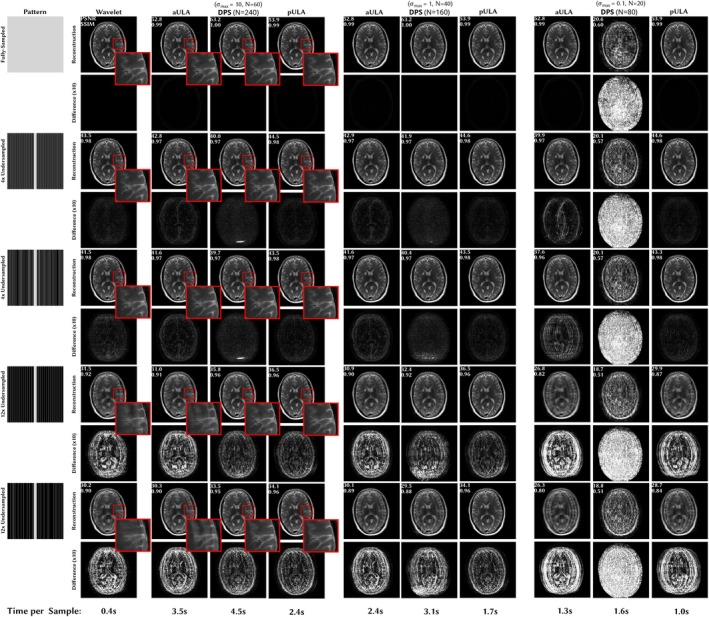
Reconstructions of a T2‐weighted brain image for different undersampling patterns using $\ell_{1}$‐Wavelet regularization and diffusion posterior sampling with annealed (pULA) and exact (ULA) likelihood. For $\ell_{1}$‐Wavelet and $\sigma_{\max}=10$ reconstruction a zoomed‐in view for a high‐detail region (highlighted in a red box) is shown. Error maps and PSNR/SSIM values are computed relative to the fully‐sampled $\ell_{1}$‐Wavelet reconstruction. Per noise level, K=8 ULA or K=4 pULA iterations were performed.

Sampling results for the Cartesian data are shown in Figure 2. While all methods yield good reconstructions, the exact likelihood with pULA requires overall less computation time (c.f. Supporting Information for the extended analysis) and is consistently outperforming the annealed likelihood and DPS method in terms of PSNR and SSIM as well as showing reduced visible differences in the error maps. The exact likelihood approach is faster, as it requires less network evaluations compared to aULA and no backpropagation through the network as DPS does. Moreover, the fast convergence of the exact likelihood approach allows for starting at smaller noise scales ($\sigma_{\max}=0.1$ for 4× acceleration and $\sigma_{\max}=1$ for 12× acceleration), which further reduces the reconstruction time compared to the other methods, which require starting at higher noise.

**FIGURE 3:**
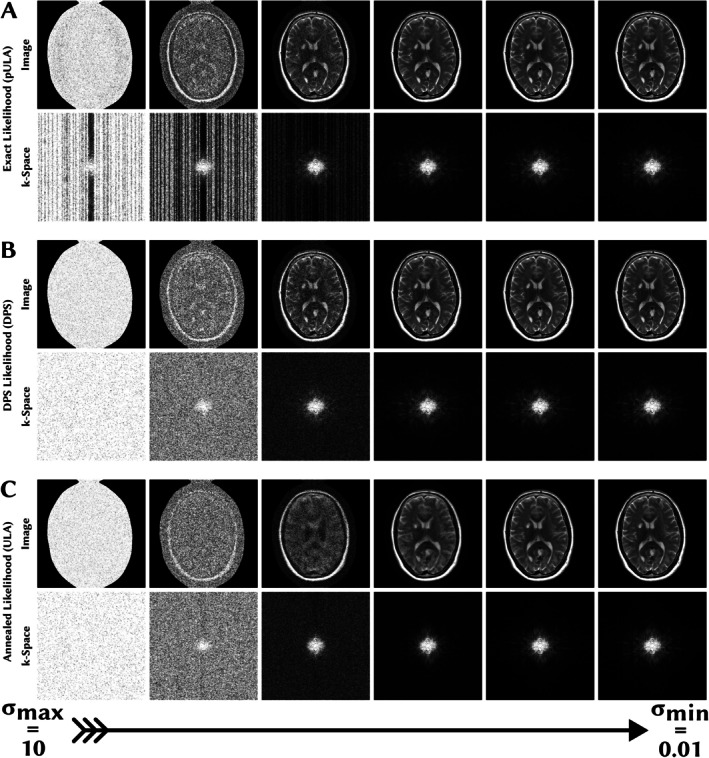
Reverse diffusion process with exact (A), DPS (B) and annealed (C) likelihood for a T2‐weighted brain image sampled from 8× random undersampled data.

The diffusion processes for 8× randomly undersampled k‐space data are shown in Figure 3. When using the exact likelihood, even at high noise levels, noise is only visible in k‐space locations that are not acquired. In contrast, for the annealed likelihood and DPS, noise appears in all k‐space lines at the beginning of the reverse diffusion process. At a high noise scale, the annealed method first generates what seems to resemble a T1‐weighted image with dark CSF, before the data term becomes dominant enough to guide the reconstruction towards the correct image. This behavior does not occur when using the exact likelihood.

**FIGURE 4:**
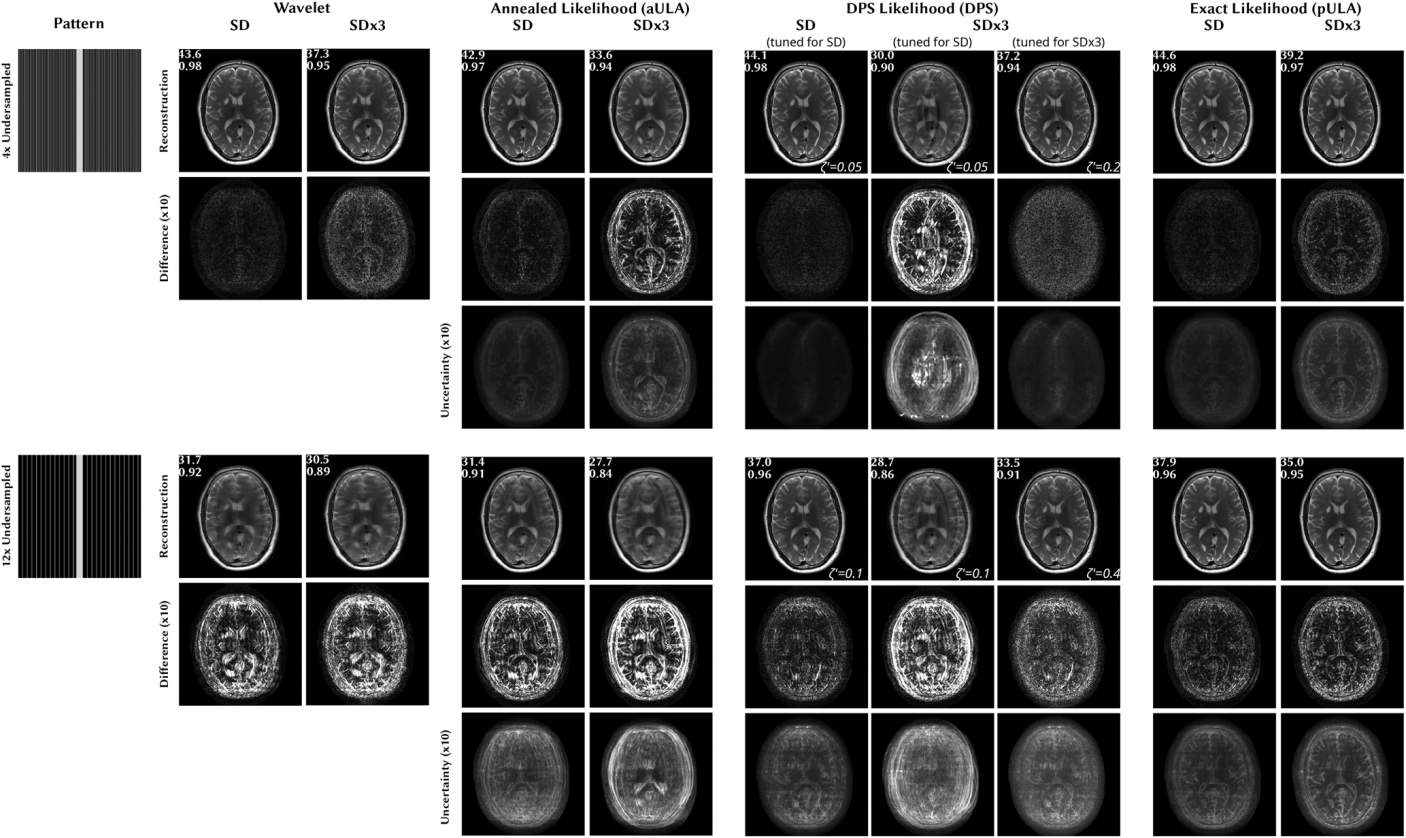
Comparison of reconstructions from original k‐space data (SD) and three‐fold increased noise level (SDx3). The weighting of the DPS likelihood ($\zeta^{\prime }$) was tuned for each noise level. Using $\zeta^{\prime }$ tuned for the wrong noise level shows a significant drop in performance. aULA and pULA adapt to the noise level without the need for tuning, and pULA yields the best results in PSNR for both noise levels.

The performance of all methods with noise‐corrupted k‐space data is presented in Figure 4. The exact likelihood approach combined with pULA delivers consistently strong results across all scenarios, including high undersampling patterns and different noise corruption. Notably, DPS requires tuning of the likelihood weighting $\zeta^{\prime }$ for each noise level to achieve good performance (c.f. Figure S4). Reconstructions for different numbers of virtual coils are shown in Figure S5.

**FIGURE 5:**
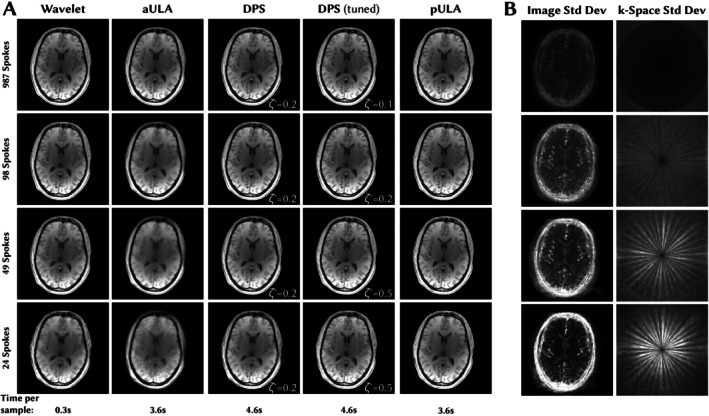
Brain image reconstructed from radially acquired FLASH data using different undersampling factors. A: Reconstruction with $\ell_{1}$‐Wavelet regularization, the average of ten samples from the posterior drawn with annealed likelihood (aULA), DPS, and the exact likelihood (pULA). DPS was once performed with the weighting $\zeta^{\prime }=0.2$ from the Cartesian experiments and once with $\zeta^{\prime }$ tuned per undersampling. B: Pixel‐wise standard deviation map of the exact likelihood approach in image space and k‐space.

The results of the non‐Cartesian reconstruction are shown in Figure 5. Reconstructions with the annealed likelihood are blurred due to the ill‐conditioning of the non‐Cartesian SENSE model, even when all spokes are used. The DPS approach shows comparable results to the exact likelihood approach, however, only for specific weightings $\zeta^{\prime }$ tuned for each undersampling factor (c.f. Figure S6). The pixel‐wise standard deviation shows high uncertainty in regions containing small vessels.

## 5 Discussion

In this work, we addressed the problem that existing methods for posterior sampling for MRI reconstruction are rather slow and necessitate cumbersome parameter tuning to balance computation time and errors depending on the ill‐posedness of the inverse problem. We investigated the use of an exact likelihood term rather than adjusting the likelihood in the diffusion process as done in previous publications.

In a 2D toy example, one can see that a naive use of the exact likelihood leads to slow convergence because the data restricts the problem space along one dimension, preventing large step sizes. While the annealed likelihood avoids this problem as it reduces the maximum eigenvalue, it comes at the cost of not utilizing the data properly at later diffusion times and requires careful parameter tuning. As we showed, slow convergence can be addressed effectively by preconditioning. The exact likelihood with pULA then shows similar performance in the 2D toy example as the annealed likelihood method. This observation can be extended to real MRI data where sampling with exact likelihood and pULA then consistently outperforms the annealing and DPS, and shows robust behavior for different undersampling masks, numbers of virtual coils, and non‐Cartesian radial MRI while using the same step size, number of iterations, and the same maximum noise level in all scenarios.

Similar to other posterior sampling methods, the fundamental assumption of the exact likelihood is that the MRI measurement is accurately described by the SENSE forward model and additive Gaussian noise. In case of a model mismatch, e.g., due to motion or spike noise, it may be beneficial to adapt the forward model to include motion [34] or to adaptively reweight the data consistency term to respect potential outliers in case of spike noise [35].

As our results show, the exact likelihood approach with pULA is significantly faster than the annealed or DPS approach, while achieving the same or superior reconstruction quality. This is because fewer expensive network evaluations are required for convergence. Both, network evaluations and CG steps scale (log)‐linearly with the problem size, such that we expect similar benefits of the exact likelihood approach with pULA for 3D reconstructions. However, poorer problem conditioning for 3D‐non‐Cartesian sampling could require more CG iterations for preconditioning.

Using the exact likelihood naturally restricts sampling to the problem space determined by the data and eliminates the need for likelihood approximation or hyperparameter tuning. Although this formulation requires small step sizes in practice, this limitation can be effectively mitigated through preconditioning. In this sense, the exact likelihood approach combined with pULA constitutes a unified method that enables efficient and principled sampling from the posterior distribution, while automatically adapting to different acquisition settings and measurement noise levels without requiring hyperparameter tuning.

## 6 Conclusion

The proposed exact likelihood with preconditioned ULA enables fast and robust posterior sampling for different MRI reconstruction problems without parameter tuning. Especially ill‐conditioned problems such as radial MRI benefit from the increased convergence speed.

## Funding

The research was funded in whole or in part by the Austrian Science Fund (FWF) 10.55776/F100800 and National Science Foundation (NSF) CCF‐2239687 (CAREER).

## Conflicts of Interest

The authors declare no conflicts of interest.

## Supporting information


**Figure S1:** Comparison of different DPS likelihood weightings ζ' for the DPS method in Figure 2. The optimal weighting interms of PSNR is highlighted in yellow. It depends on the acceleration factor.
**Figure S2:** Comparison of annealed likelihood and exact likelihood with pULA for different numbers of Langevin iterations K per noise level, and using different numbers of CG iterations *N*_*CG*_. Reconstruction times are per sample. The quantitative metrics and intensity of the difference maps suggest that pULA with K = 1 and *N*_*CG*_ = 8 reaches similar reconstruction quality as the annealed likelihood with *K* = 100 and outperforms it for *K* = 8, whereas the reconstruction time is significantly reduced by pULA. Further, increasing the number of CG iterations to *N*_*CG*_ = 50 does not lead to visible changes in reconstruction quality, but slightly decreases the PSNR, potentially due to numerical inaccuracies.
**Figure S3:** Measured reconstruction time of pULA depending on the number of CG and Langevin iterations per noise level. The measured times are presented as dots, whereas the solid lines represent linear fits using the model in Equation 26. The fitted parameters are t_Network_≈4.5 ms, t_AHA_≈0.13 ms and t_*ini*_≈3.1 s. The large t_*ini*_, is mostly due to initialization of the GPU and the network, but can be amortized when many reconstructions are performed. The evaluation of the neural network is the dominant cost, whereas the cost for applying *A* ^*H*^ *A* is about 34 times lower.
**Figure S4:** Comparison of different DPS likelihood weightings ζ' for the DPS method in Figure 4. Highlighting that the optimal step size ξ' for different undersampling pattern and noise corruption.
**Figure S5:** Reconstructions of a T1‐weighted brain image for selected undersampling patterns and different numbers of virtual coils after coil compression. Uncertainty maps show the standard deviation over drawn samples.
**Figure S6:** Comparison of different DPS likelihood weightings ζ' for the DPS method in Figure 4. Highlighting that the optimal step size ξ' depends on the number of spokes.

## Data Availability

In the spirit of reproducible research, the code to reproduce the results of this paper is available at https://gitlab.tugraz.at/ibi/mrirecon/papers/dps‐pula (Version v0.2). All reconstructions have been performed with BART (v1.0.00), available at https://codeberg.org/mrirecon/bart. The data used in this study is available at Zenodo (DOI: 10.5281/zenodo.17739731).

## References

[1] A. Jalal , M. Arvinte , G. Daras , E. Price , A. G. Dimakis , and J. Tamir , “Robust Compressed Sensing MRI With Deep Generative Priors,” in Advances in Neural Information Processing Systems, vol. 34 (Curran Associates, Inc, 2021), 14938–14954.

[2] H. Chung and J. C. Ye , “Score‐Based Diffusion Models for Accelerated MRI,” Medical Image Analysis 80 (2022): 102479. 35696876 10.1016/j.media.2022.102479

[3] G. Luo , M. Blumenthal , M. Heide , and M. Uecker , “Bayesian MRI Reconstruction With Joint Uncertainty Estimation Using Diffusion Models,” Magnetic Resonance in Medicine 90 (2023): 295–311. 36912453 10.1002/mrm.29624

[4] O. Schad , J. F. Heidenreich , N. Petri , et al., “A Spatio‐Temporal Diffusion Model for Cardiac Real‐Time Imaging,” Magnetic Resonance in Medicine 95 (2026): 3574–3583. 41708490 10.1002/mrm.70303 PMC13049260

[5] Y. Song and S. Ermon , “Generative Modeling by Estimating Gradients of the Data Distribution,” in Advances in Neural Information Processing Systems, vol. 32 (Curran Associates, Inc, 2019).

[6] J. Ho , A. Jain , and P. Abbeel , “Denoising Diffusion Probabilistic Models,” in Advances in Neural Information Processing Systems, vol. 33 (Curran Associates, Inc, 2020), 6840–6851.

[7] T. Karras , M. Aittala , T. Aila , and S. Laine , “Elucidating the Design Space of Diffusion‐Based Generative Models,” in Advances in Neural Information Processing Systems, vol. 35 (Curran Associates, Inc, 2022), 26565–26577.

[8] K. P. Pruessmann , M. Weiger , M. B. Scheidegger , and P. Boesiger , “SENSE: Sensitivity Encoding for Fast MRI,” Magnetic Resonance in Medicine 42 (1999): 952–962. 10542355

[9] A. Güngör , B. B. Bilecen , and T. Çukur , “Bayesian Conditioned Diffusion Models for Inverse Problems,” arXiv (2024), 10.48550/arXiv.2406.09768.

[10] G. Daras , H. Chung , C. H. Lai , et al., “A Survey on Diffusion Models for Inverse Problems,” arXiv (2024), 10.48550/arXiv.2410.00083.

[11] H. Chung , J. Kim , and J. C. Ye , “Diffusion Models for Inverse Problems,” arXiv (2025), 10.48550/arXiv.2508.01975.

[12] H. Chung , J. Kim , M. T. Mccann , M. L. Klasky , and J. C. Ye , “Diffusion Posterior Sampling for General Noisy Inverse Problems,” ICLR 2023: The Eleventh International Conference on Learning Representations 11 (2023).

[13] Y. Janati , E. Moulines , J. Olsson , and A. Oliviero‐Durmus , “Bridging Diffusion Posterior Sampling and Monte Carlo Methods: A Survey,” Philosophical Transactions of the Royal Society A 383 (2025): 20240331. 10.1098/rsta.2024.0331 40534298

[14] H. Chung , S. Lee , and J. C. Ye , “Decomposed Diffusion Sampler for Accelerating Large‐Scale Inverse Problems,” in The Twelfth International Conference on Learning Representations (openreview.net, 2024), https://openreview.net/forum?id=DsEhqQtfAG.

[15] K. Kreutz‐Delgado , “The Complex Gradient Operator and the CR‐Calculus,” *arXiv* (2009), 10.48550/ARXIV.0906.4835.

[16] T. Holliber , M. Blumenthal , and M. Uecker , “Unadjusted Langevin Sampling for Uncertainty Estimation in MRI Reconstruction ‐ Theory and Numerical Validation,” in Proceedings of the 2025 ISMRM & ISMRT (2025), 2603.

[17] A. S. Dalalyan , “Theoretical Guarantees for Approximate Sampling From Smooth and Log‐Concave Densities,” Journal of the Royal Statistical Society, Series B: Statistical Methodology 79 (2017): 651–676.

[18] B. Kawar , M. Elad , S. Ermon , and J. Song , “Denoising Diffusion Restoration Models,” in Advances in Neural Information Processing Systems, vol. 35 (Curran Associates, Inc, 2022), 23593–23606.

[19] J. Sohl‐Dickstein , E. A. Weiss , N. Maheswaranathan , and S. Ganguli , “Deep Unsupervised Learning Using Nonequilibrium Thermodynamics,” in Proceedings of the 32nd International Conference on Machine Learning, vol. 37 (PMLR, 2015), 2256–2265.

[20] G. O. Roberts and O. Stramer , “Langevin Diffusions and Metropolis‐Hastings Algorithms,” Methodology and Computing in Applied Probability 4 (2002): 337–357.

[21] M. C. Corbineau , D. Kouamé , E. Chouzenoux , J. Y. Tourneret , and J. C. Pesquet , “Preconditioned PULA for Joint Deconvolution‐Segmentation of Ultrasound Images,” IEEE Signal Processing Letters 26 (2019): 1456–1460.

[22] Y. Marnissi , E. Chouzenoux , A. Benazza‐Benyahia , and J. C. Pesquet , “Majorize–Minimize Adapted Metropolis–Hastings Algorithm,” IEEE Transactions on Signal Processing 68 (2020): 2356–2369.

[23] R. Bhattacharya and T. Jiang , “Fast Sampling and Inference via Preconditioned Langevin Dynamics,” *arXiv* (2024), 10.48550/arXiv.2310.07542.

[24] G. Papandreou and A. L. Yuille , “Gaussian Sampling by Local Perturbations,” in Advances in Neural Information Processing Systems, vol. 23 (Curran Associates, Inc, 2010).

[25] H. Robbins , “An Empirical Bayes Approach to Statistics,” Proceedings of the Third Berkeley Symposium on Mathematical Statistics and Probability (University of California Press, 1954), 157–163.

[26] F. Knoll , J. Zbontar , A. Sriram , et al., “fastMRI: A Publicly Available Raw k‐Space and DICOM Dataset of Knee Images for Accelerated MR Image Reconstruction Using Machine Learning. *Radiology* ,” Artificial Intelligence 2 (2020): e190007. 32076662 10.1148/ryai.2020190007 PMC6996599

[27] M. Uecker , T. Hohage , K. T. Block , and J. Frahm , “Image Reconstruction by Regularized Nonlinear Inversion‐Joint Estimation of Coil Sensitivities and Image Content,” Magnetic Resonance in Medicine 60 (2008): 674–682. 18683237 10.1002/mrm.21691

[28] M. Uecker , P. Lai , M. J. Murphy , et al., “ESPIRiT—An Eigenvalue Approach to Autocali‐Brating Parallel MRI: Where SENSE Meets GRAPPA,” Magnetic Resonance in Medicine 71 (2014): 990–1001. 23649942 10.1002/mrm.24751 PMC4142121

[29] Y. Song , J. Sohl‐Dickstein , D. P. Kingma , A. Kumar , S. Ermon , and B. Poole , “Score‐Based Generative Modeling Through Stochastic Differential Equations,” in ICLR 2021: The Ninth International Conference on Learning Representations, vol. 9 (openreview.net, 2021), https://openreview.net/forum?id=PxTIG12RRHS.

[30] M. Blumenthal , G. Luo , M. Schilling , H. C. M. Holme , and M. Uecker , “Deep, Deep Learning With BART,” Magnetic Resonance in Medicine 89 (2023): 678–693. 36254526 10.1002/mrm.29485 PMC10898647

[31] B. Efron , “Tweedie's Formula and Selection Bias,” Journal of the American Statistical Association 106 (2011): 1602–1614. 22505788 10.1198/jasa.2011.tm11181 PMC3325056

[32] N. Scholand , P. Schaten , C. Graf , et al., “Rational Approximation of Golden Angles: Accelerated Reconstructions for Radial MRI,” Magnetic Resonance in Medicine 93 (2025): 51–66. 39250418 10.1002/mrm.30247 PMC12034029

[33] S. Rosenzweig , H. C. M. Holme , and M. Uecker , “Simple Auto‐Calibrated Gradient Delay Estimation From Few Spokes Using Radial Intersections (RING),” Magnetic Resonance in Medicine 81 (2019): 1898–1906. 30489652 10.1002/mrm.27506

[34] B. Levac , S. Kumar , A. Jalal , and J. I. Tamir , “Accelerated Motion Correction With Deep Generative Diffusion Models,” Magnetic Resonance in Medicine 92 (2024): 853–868. 38688874 10.1002/mrm.30082

[35] L. Hen , T. Tirer , R. Giryes , and S. Abu‐Hussein , “Robust Posterior Diffusion‐Based Sampling via Adaptive Guidance Scale,” arXiv (2025), 10.48550/arXiv.2511.18471.

