# Supplementary material for: Fast and Robust Diffusion Posterior Sampling for MR Image Reconstruction Using the Preconditioned Unadjusted Langevin Algorithm
Source: Magn Reson Med. 2026 May 10;96(3):1323–32. doi: 10.1002/mrm.70416 (PMC13327450; doi:10.1002/mrm.70416)
Supplement: Supplementary file 1 — Figure S1: Comparison of different DPS likelihood weightings ζ' for the DPS method in Figure 2. The optimal weighting interms of PSNR is highlighted in yellow. It depends on the acceleration factor. Figure S2: Comparison of annealed likelihood and exact likelihood with pULA for different numbers of Langevin iterations K per noise level, and using different numbers of CG iterations N CG . Reconstruction times are per sample. The quantitative metrics and intensity of the difference maps suggest that pULA with K = 1 and N CG = 8 reaches similar reconstruction quality as the annealed likelihood with K = 100 and outperforms it for K = 8, whereas the reconstruction time is significantly reduced by pULA. Further, increasing the number of CG iterations to N CG = 50 does not lead to visible changes in reconstruction quality, but slightly decreases the PSNR, potentially due to numerical inaccuracies. Figure S3: Measured reconstruction time of pULA depending on the number of CG and Langevin iterations per noise level. The measured times are presented as dots, whereas the solid lines represent linear fits using the model in Equation 26. The fitted parameters are tNetwork≈4.5 ms, tAHA≈0.13 ms and t ini ≈3.1 s. The large t ini , is mostly due to initialization of the GPU and the network, but can be amortized when many reconstructions are performed. The evaluation of the neural network is the dominant cost, whereas the cost for applying A H A is about 34 times lower. Figure S4: Comparison of different DPS likelihood weightings ζ' for the DPS method in Figure 4. Highlighting that the optimal step size ξ' for different undersampling pattern and noise corruption. Figure S5: Reconstructions of a T1‐weighted brain image for selected undersampling patterns and different numbers of virtual coils after coil compression. Uncertainty maps show the standard deviation over drawn samples. Figure S6: Comparison of different DPS likelihood weightings ζ' for the DPS method in Figure 4. Highlighti [file MRM-96-1323-s001.pdf]

Supporting Information

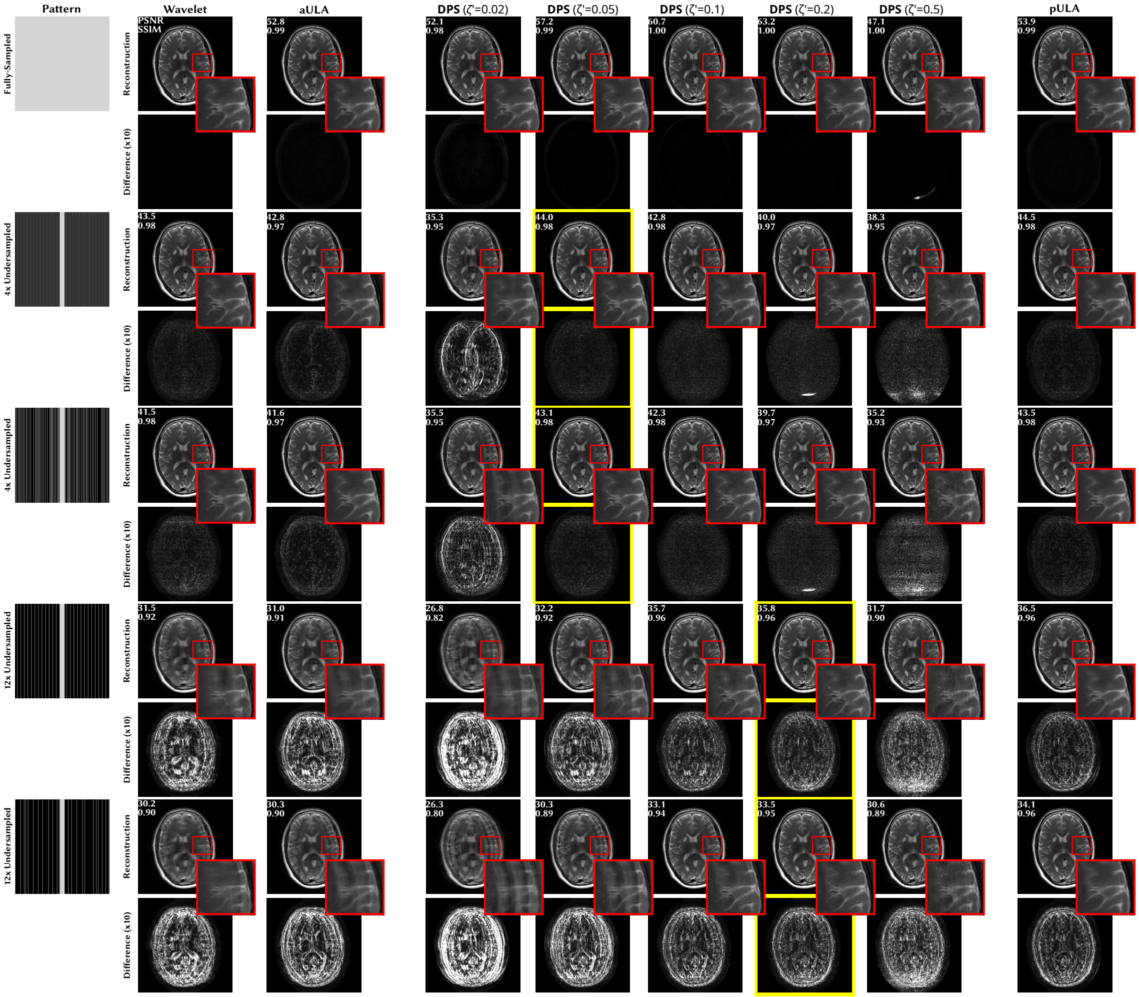

Figure S1: Comparison of different DPS likelihood weightings ζ' for the DPS method in Figure 2. The optimal weighting in terms of PSNR is highlighted in yellow. It depends on the acceleration factor.

S1 Exact Likelihood and Preconditioning Matrix in Variance Preserving Formulation

In the variance exploding (VE) formulation of diffusion models, the smoothed prior distributions are defined by convolution with a Gaussian smoothing kernel, i.e.

$$p_t(x_t|x_0) = \mathcal{CN}(x_t; x_0, \sigma_t^2 I)\,. \tag{14}$$

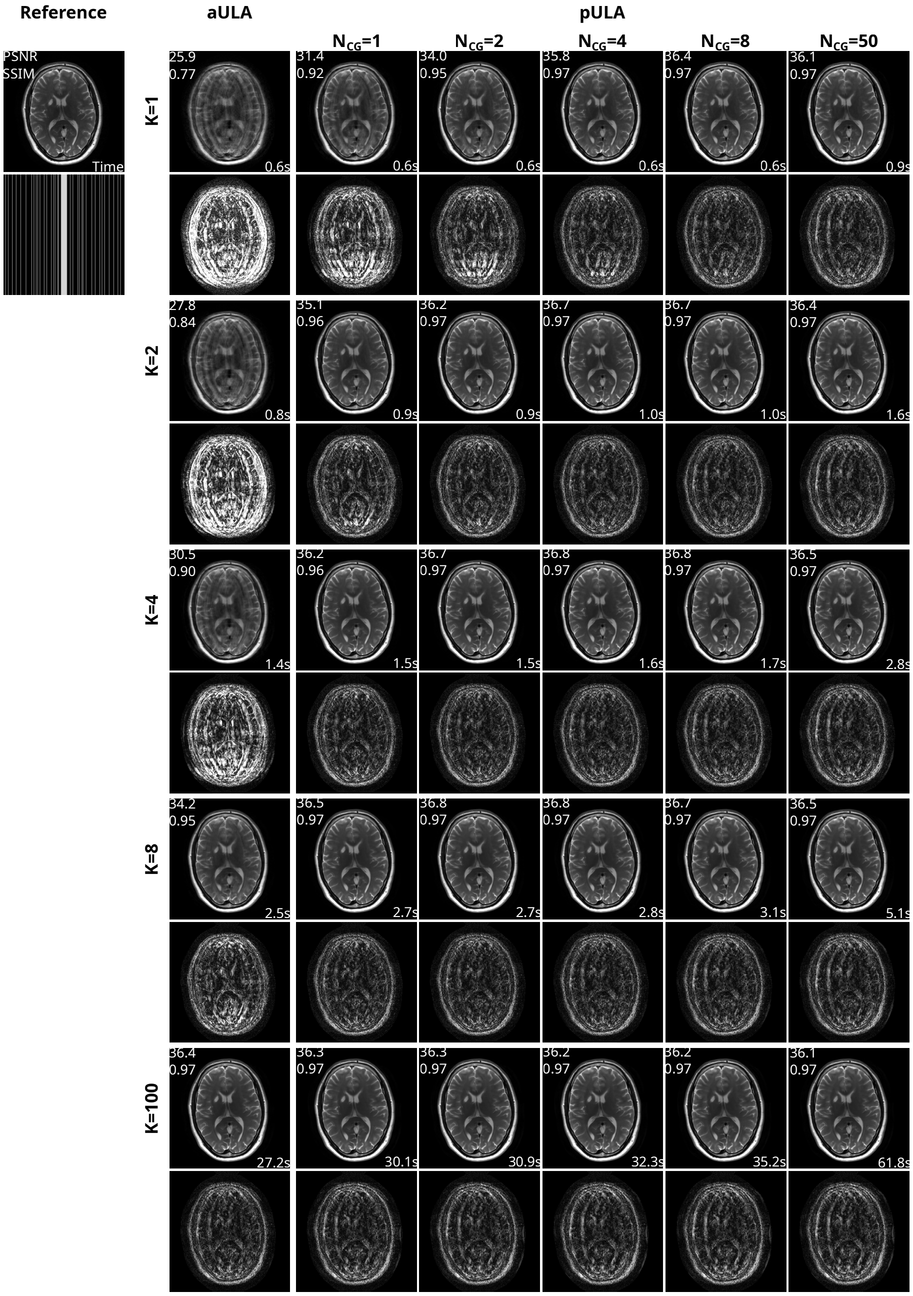

Figure S2: Comparison of annealed likelihood and exact likelihood with pULA for different numbers of Langevin iterations K per noise level, and using different numbers of CG iterations N_{CG}. Reconstruction times are per sample. The quantitative metrics and intensity of the difference maps suggest that pULA with $K = 1$ and $N_{CG} = 8$ reaches similar reconstruction quality as the annealed likelihood with $K = 100$ and outperforms it for $K = 8$, whereas the reconstruction time is significantly reduced by pULA. Further, increasing the number of CG iterations to $N_{CG} = 50$ does not lead to visible changes in reconstruction quality, but slightly decreases the PSNR, potentially due to numerical inaccuracies.

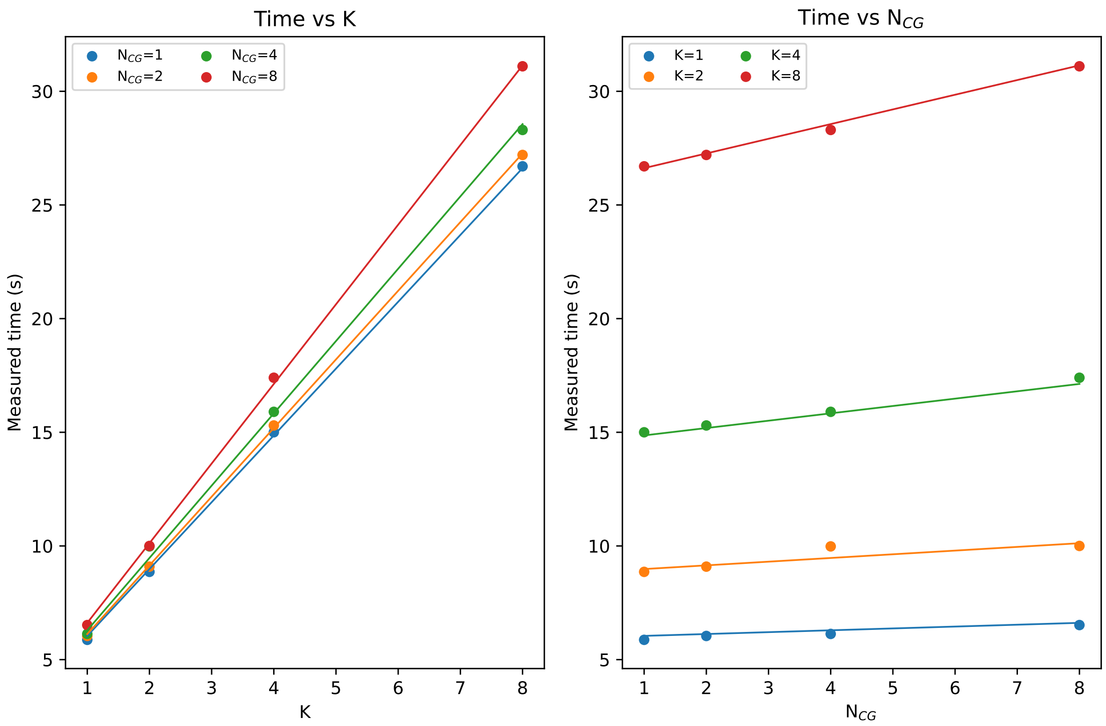

Figure S3: Measured reconstruction time of pULA depending on the number of CG and Langevin iterations per noise level. The measured times are presented as dots, whereas the solid lines represent linear fits using the model in Eq. 26. The fitted parameters are $t_{\mathrm{Network}} \approx 4.5\,\mathrm{ms}$, $t_{A^H A} \approx 0.13\,\mathrm{ms}$ and $t_{ini} \approx 3.1\,\mathrm{s}$. The large t_{ini}, is mostly due to initialization of the GPU and the network, but can be amortized when many reconstructions are performed. The evaluation of the neural network is the dominant cost, whereas the cost for applying $A^H A$ is about 34 times lower.

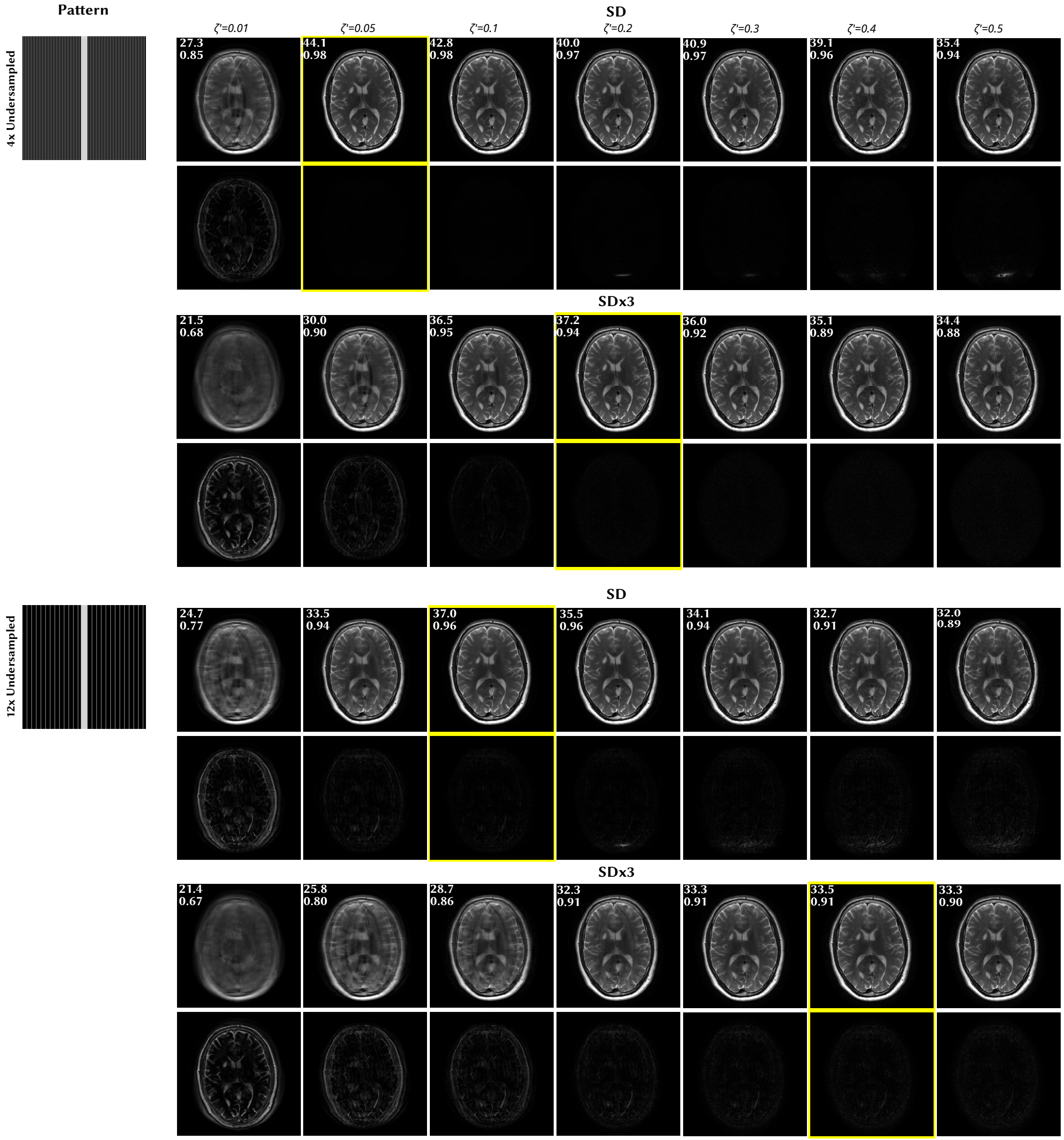

Figure S4: Comparison of different DPS likelihood weightings ζ' for the DPS method in Figure 4. Highlighting that the optimal step size ξ' for different undersampling pattern and noise corruption.

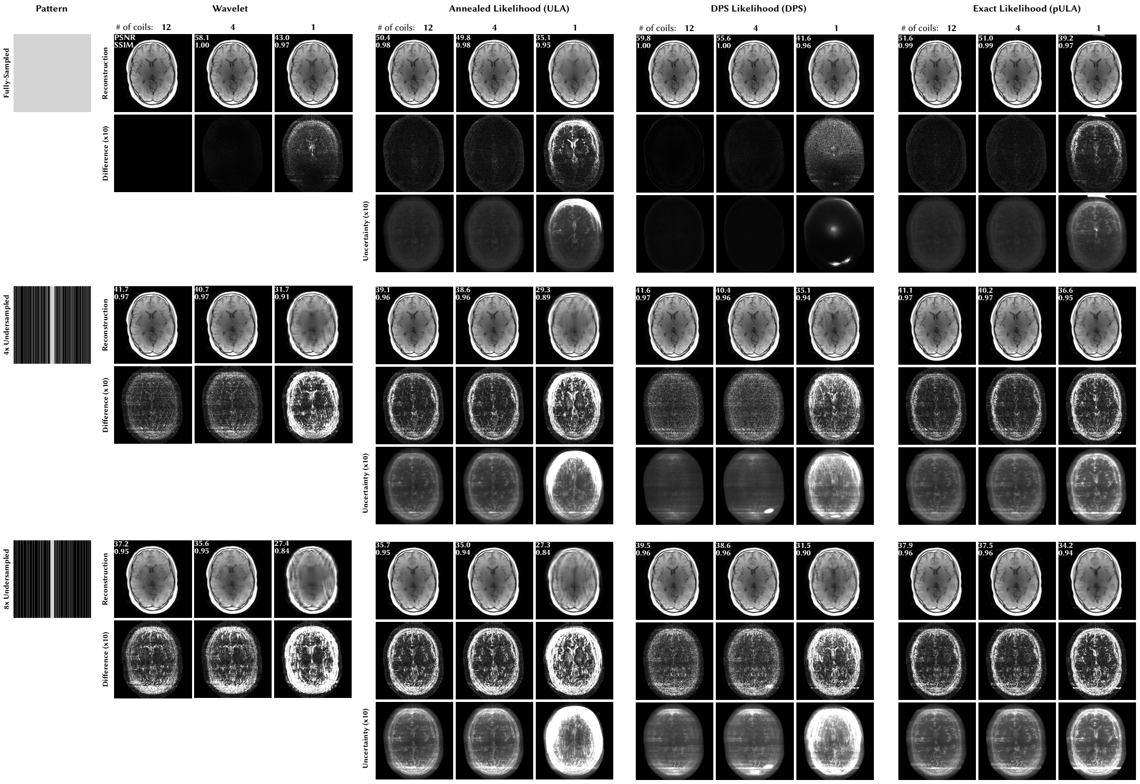

Figure S5: Reconstructions of a T1-weighted brain image for selected undersampling patterns and different numbers of virtual coils after coil compression. Uncertainty maps show the standard deviation over drawn samples.

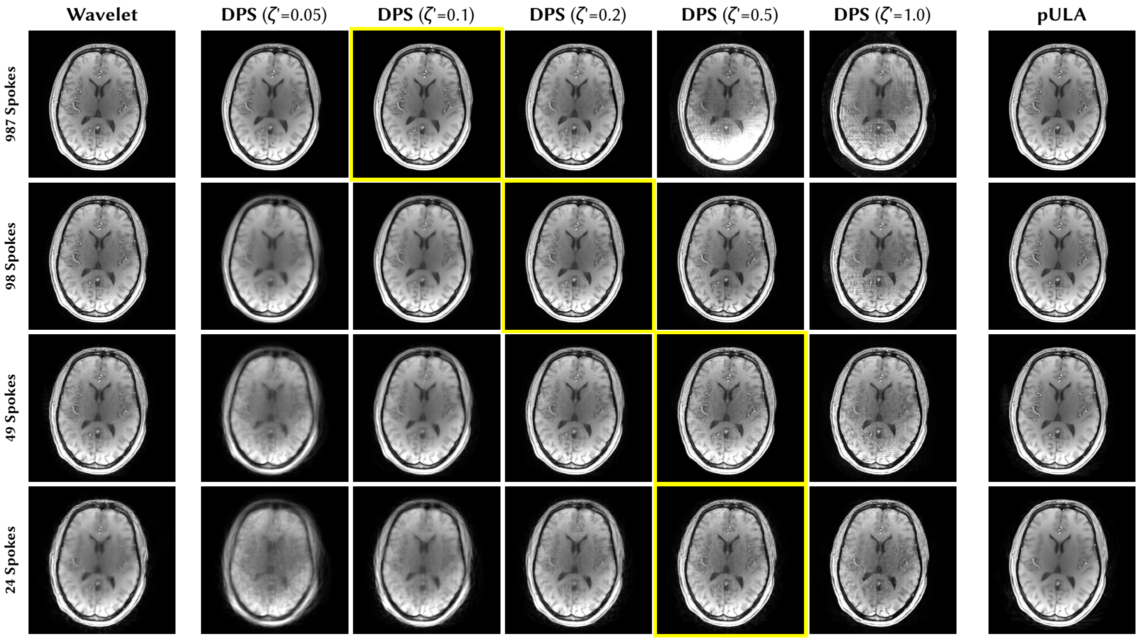

Figure S6: Comparison of different DPS likelihood weightings ζ' for the DPS method in Figure 4. Highlighting that the optimal step size ξ' depends on the number of spokes.

In contrast, in the variance preserving (VP) formulation, the perturbation kernel reads

$$\tilde{p}_t(\tilde{x}_t|x_0) = \mathcal{CN}\left(\tilde{x}_t; \alpha_t x_0, (1-\alpha_t^2)I\right) \,, \tag{15}$$

where we us $\tilde{\cdot}$ to distinguish variables in the VP formulation from those in the VE formulation. Both formulations are equivalent by identifying

$$x_t = \frac{\tilde{x}_t}{\alpha_t} \qquad \text{and} \qquad \sigma_t^2 = \frac{1-\alpha_t^2}{\alpha_t^2} \,. \tag{16}$$

The *Exact Likelihood* term from Eq. 6 reads in the VP formulation

$$\tilde{p}^{\text{exact}}(\mathbf{y}|\tilde{\mathbf{x}}_t) \propto \exp\left(-\|\mathbf{y} - \alpha_t^{-1}A\tilde{\mathbf{x}}_t\|_2^2\right) , \tag{17}$$

yielding a ULA update step of

$$\tilde{\mathbf{x}}_t^{k+1} = \tilde{\mathbf{x}}_t^k + \gamma\left[\alpha_t^{-1}A^H(\mathbf{y} - \alpha_t^{-1}A\tilde{\mathbf{x}}_t^k) + \nabla_{\tilde{\mathbf{x}}}\log\tilde{p}_t(\tilde{\mathbf{x}}_t^k)\right] + \sqrt{2\gamma}\mathbf{z}^k \qquad \mathbf{z}^k \sim \mathbb{CN}(\mathbf{0}, \mathbb{I}) \,. \tag{18}$$

By reparametrization the Hessian of the log-prior transforms with α_t^2 and, hence, the inverse preconditioning matrix in the VP formulation is given by

$$\tilde{M}_t^{-1} = \alpha_t^{-2}A^HA + \alpha_t^{-2}\sigma_t^{-2}\mathbb{I} = \alpha_t^{-2}A^HA + \left(1-\alpha_t^2\right)^{-1}\mathbb{I} \,. \tag{19}$$

S2 Warmstart of Conjugate Gradient Algorithm

To reduce the number of CG iterations when applying the preconditioning matrix, we warmstart the CG algorithm. For this we first note that the pULA update as expressed in the main manuscript can be rewritten as

$$\begin{aligned}
\mathbf{x}_t^{k+1} &= \mathbf{x}_t^k + \gamma M_t\left[A^H\left(\mathbf{y} + \sqrt{\frac{2}{\gamma}}\mathbf{n}_1^k - A\mathbf{x}_t^k\right) + \nabla_{\mathbf{x}}\log p(\mathbf{x}_t^k) + \sqrt{\frac{2}{\gamma\sigma_t^2}}\mathbf{n}_2^k\right] \\
&= \mathbf{x}_t^k + M_t\left[A^H\left(\gamma\mathbf{y} + \sqrt{2\gamma}\mathbf{n}_1^k - A\gamma\mathbf{x}_t^k\right) + \gamma\nabla_{\mathbf{x}}\log p(\mathbf{x}_t^k) + \sqrt{\frac{2\gamma}{\sigma_t^2}}\mathbf{n}_2^k\right] \\
&= M_t\left[A^H\left(\gamma\mathbf{y} + \sqrt{2\gamma}\mathbf{n}_1^k - A\gamma\mathbf{x}_t^k\right) + \gamma\nabla_{\mathbf{x}}\log p(\mathbf{x}_t^k) + \sqrt{\frac{2\gamma}{\sigma_t^2}}\mathbf{n}_2^k + A^HA\mathbf{x}_t^k + \sigma_t^{-2}\mathbf{x}_t^k\right] \,.
\end{aligned} \tag{20}$$

In this form, the pULA update is directly given by the solution of the CG algorithm. We initialize this conjugate gradient algorithm with

$$\mathbf{x}_{t,\text{init}}^{k+1} = \mathbf{x}_t^k + \gamma\sigma_t^2\nabla_{\mathbf{x}}\log p(\mathbf{x}_t^k) + \sqrt{2\gamma\sigma_t^2}\mathbf{n}_2^k \,, \tag{21}$$

corresponding to an update step only using the prior score. Supporting Figure S2 shows, that using this warmstart strategy, already one CG iteration leads to improved reconstructions compared to the annealed likelihood method.

S3 Implementation of DPS in the Variance Exploding Formulation

The DPS [11] method approximates the diffused likelihood by

$$p^{\mathrm{DPS}}(\mathbf{y}|\mathbf{x}_t) \propto \exp\left(-\|\mathbf{y} - A\mathbb{E}[\mathbf{x}_0|\mathbf{x}_t]\|_2^2\right). \tag{22}$$

We have implemented DPS in the variance exploding formulation by integrating the corresponding reverse stochastic differential equation using the predictor approach described in Algorithm 2 of [28]. Using N steps to discretize the time t and denoting $\sigma_i = \sigma(i/N)$ the respective noise levels, DPS likelihood from Eq. 22 into the predictor update step yields

$$\begin{aligned} \mathbf{x}_i = \mathbf{x}_{i+1} &+ \left(\sigma_{i+1}^2 - \sigma_i^2\right)\left[\nabla_{\bar{\mathbf{x}}} \log p(\mathbf{x}_{i+1}) + \nabla_{\bar{\mathbf{x}}} \log p^{\mathrm{DPS}}(\mathbf{y}|\mathbf{x}_{i+1})\right] \\ &+ \sqrt{\sigma_{i+1}^2 - \sigma_i^2}\,\mathbf{z}_i \qquad \mathbf{z}_i \sim \mathbb{C}\mathcal{N}(\mathbf{0}, \mathbb{I}). \end{aligned} \tag{23}$$

It was observed by Chung et al. [11] that using equal weighting of the likelihood and the prior score leads to suboptimal results, such that they introduced a weighting parameter ζ to balance the two terms. Heuristically, the weighting was chosen based on the current residual. We set it to

$$\zeta_{i+1} = \frac{\zeta'}{\left(\sigma_{i+1}^2 - \sigma_i^2\right)\|\mathbf{y} - A\mathbb{E}[\mathbf{x}_0|\mathbf{x}_{i+1}]\|}, \tag{24}$$

where ζ' is a hyperparameter that needs to be tuned. The implemented update step for our DPS implementation reads

$$\begin{aligned} \mathbf{x}_i = \mathbf{x}_{i+1} &+ \left(\sigma_{i+1}^2 - \sigma_i^2\right)\left[\nabla_{\bar{\mathbf{x}}} \log p(\mathbf{x}_{i+1}) + \zeta_{i+1}\nabla_{\bar{\mathbf{x}}} \log p^{\mathrm{DPS}}(\mathbf{y}|\mathbf{x}_{i+1})\right] \\ &+ \sqrt{\sigma_{i+1}^2 - \sigma_i^2}\,\mathbf{z}_i \qquad \mathbf{z}_i \sim \mathbb{C}\mathcal{N}(\mathbf{0}, \mathbb{I}). \end{aligned} \tag{25}$$

S4 Runtime Analysis of aULA and pULA

Methods

The computational complexity of pULA was investigated by varying the number of Langevin iterations $K = 1, 2, 4, 8$ and CG iterations $N_{CG} = 1, 2, 4, 8$. The expected dominant computational cost of aULA are the score network evaluation (t_{Network}) and the normal operator ($t_{A^H A}$), once in each Langevin iteration. For pULA, $N_{CG} + 1$ additional applications of $A^H A$ are required in each Langevin iteration for the preconditioning and $N_{CG} + 1$ evaluations for the initialization. Hence, the total reconstruction time t_{total} is modeled as

$$t_{\mathrm{total}} = N_S N K \cdot t_{\mathrm{Network}} + N_S N K (N_{CG} + 1) \cdot t_{A^H A} + (N_{CG} + 1) \cdot t_{A^H A} + t_{\mathrm{init}}, \tag{26}$$

where t_{init} summarizes all additional one-time computations such as initialization of the GPU and the score network. We fitted this model to the measured reconstruction times using least squares to estimate t_{Network}, t_{A^HA}, and t_{init}.

Results

Reconstruction results for different numbers of Langevin and CG iterations are shown in Supporting Figure S2. It shows that by using preconditioning, the number of expensive network applications can be reduced without loss in reconstruction quality, whereas increasing the number of CG iterations beyond 8 does not lead to further significant improvements.

The measured reconstruction times as functions of the number of Langevin and CG iterations are shown in Supporting Figure S3, together with the fitted model from Eq. 26. The fitted parameters are $t_{\text{Network}} \approx 4.5\,\text{ms}$, $t_{A^HA} \approx 0.13\,\text{ms}$ and $t_{ini} \approx 3.1\,\text{s}$. It confirms that in our setting the cost for applying the network is the dominant cost whereas applying A^HA is about 34 times lower.
